# Prevalence and Molecular Epidemiology of Nontyphoidal *Salmonella enterica* Serovars Enteritidis and Typhimurium in Bushmeat Carcasses Sold in the Centre Region Markets, Cameroon

**DOI:** 10.1002/mbo3.70441

**Published:** 2026-10-28

**Authors:** Marius Dongmo, Akindeh Mbuh Nji, Rhoda Nsen Bughe, Calvino Fomboh Tah, Wilfred Fon Mbacham, Sylvain Leroy Sado Kamdem

**Affiliations:** ^1^ Department of Microbiology University of Yaoundé 1 Yaoundé Cameroon; ^2^ Food and Drug Safety Unit (FODRUS), Laboratory for Public Health Research Biotechnologies, Biotechnology Center, Nkolbisson University of Yaoundé 1 Yaoundé Cameroon; ^3^ Department of Biochemistry University of Yaoundé 1 Yaoundé Cameroon; ^4^ Department of Biological Sciences, Higher Teacher Training College University of Yaoundé 1 Yaoundé Cameroon

**Keywords:** bushmeat, food safety, molecular epidemiology, nontyphoidal *Salmonella*, RAPD–PCR

## Abstract

*Salmonella* is a pathogen that causes gastrointestinal illness in consumers. Animal protection regulations are frequently disregarded by certain local populations, and the microbial risks associated with their consumption make this practice a serious public health concern. Highlighting these dangers may support state regulations and reduce consumer demand. Therefore, this study aimed to characterize the genetic diversity of nontyphoidal *Salmonella* isolates from bushmeat carcasses consumed in the Central Region of Cameroon. A total of 142 fresh carcass samples were collected from local markets for *Salmonella* isolation. Phenotypically identified isolates were subjected to multiplex polymerase chain reaction (PCR) targeting the InvA gene for the *Salmonella* genus, the IE‐1 gene for *Salmonella* ser. Enteritidis, and the Flic‐C gene for *Salmonella* ser. Typhimurium. For molecular typing, *S*. Enteritidis and *S*. Typhimurium isolates were further analyzed using Random Amplification of Polymorphic DNA (RAPD–PCR). Phenotypic analysis detected *Salmonella* spp. in 117 carcass samples (82.39%), and PCR confirmed 98 of these (69.01%) as true nontyphoidal *Salmonella* isolates, comprising 93 *Salmonella* ser. Enteritidis and five *Salmonella* ser. Typhimurium. Among the 98 confirmed NTS isolates, the InvA gene was detected in 45 (45.91%), and RAPD–PCR profiles were distinct for all isolates. At a similarity threshold of 80%, *Salmonella* ser. Typhimurium isolates were grouped into three clusters, while *Salmonella* ser. Enteritidis isolates were grouped into six clusters. This study shows that bushmeat is heavily contaminated with nontyphoidal *Salmonella*, predominantly *S*. Enteritidis serovars. These results may support future epidemiological research and help raise awareness of the microbial risks associated with bush meat.

## Graphical abstract

A total of 142 bushmeat samples were obtained from five localities in the Central Region of Cameroon and analyzed for the presence of Nontyphoidal *Salmonella enterica,* which showed a high prevalence, with *Salmonella* Enteritidis being the most frequently detected and exhibiting high genetic variation.

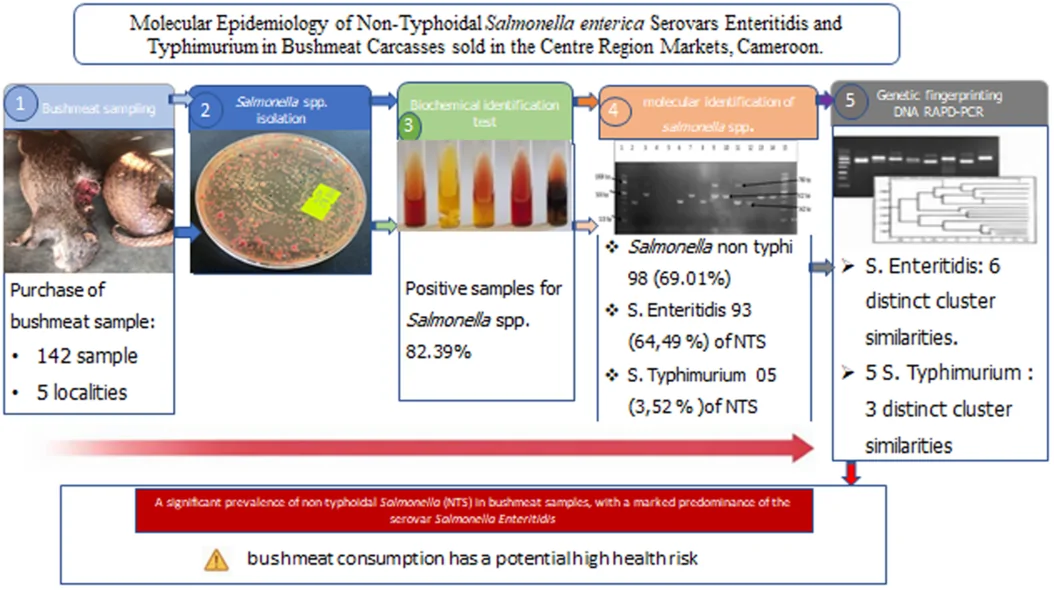

## 1 Introduction

*Salmonella* is one of the major foodborne pathogens associated with human disease and is considered a complex zoonosis with significant implications for global public health (Mendonça et al. 2020). The vast majority of human infections are caused by *Salmonella enterica* subsp. *enterica*, whose serotypes are clinically divided into typhoid and nontyphoid *Salmonella* (NTS) (Pagani et al. 2023). particularly *Salmonella* ser. Enteritidis and *Salmonella* ser. Typhimurium. Nontyphoidal is the most frequent cause of human salmonellosis worldwide (Sahu et al. 2025). They are associated domestic and wild animal reservoirs and represent one of the most common causes associated with domestic foodborne infections in both high‐income and low‐ and middle‐income countries (European Food Safety Authority and European Centre for Disease Prevention and Control 2022). Worldwide, NTS are responsible for millions of gastrointestinal infections and thousands of deaths annually (Se et al. 2010; Stanaway et al. 2019).

The primary clinical manifestation of NTS infection is enterocolitis, characterized by diarrhea, nausea, abdominal pain, and mild fever Although disease severity may range from mild self‐limiting symptoms to severe and fatal cases (Smith 2015; Souza et al. 2019). Foods of animal origin, including meat, eggs, and milk, constitute the principal sources of contamination, with poultry recognized as the major reservoir of the microorganism (Shah et al. 2017). Other reservoirs have also been identified in cattle, pigs, and wildlife species involved in disease transmission (Kagambèga et al. 2013; Soliani et al. 2023). In sub‐Saharan Africa, *Salmonella* ser. Typhimurium and *Salmonella* ser. Enteritidis are also recognized as important causes of invasive nontyphoidal *Salmonella* (iNTS) disease, particularly among children, elderly persons, HIV‐positive individuals, and malnourished populations (Gal‐Mor et al. 2014; MacLennan and Levine 2013; Gal‐Mor et al. 2014; MacLennan and Levine 2013; Mughini‐Gras et al. 2020).

In Cameroon, bushmeat consumption and commercialization remain common because a segment of the population perceives wildlife products as natural foods free from chemicals used in intensive animal farming. However, bushmeat is associated with a high risk of microbial diseases and may constitute an important reservoir of pathogenic NTS (Brittain et al. 2022). These wild animals, which do not receive antibiotic treatment throughout their lives, may be an important reservoir for iNTS. In addition, several surveys have shown that many consumers do not perceive the zoonotic risks associated with bushmeat, despite evidence of transmission during the COVID‐19 pandemic (Saylors et al. 2021). Poor hygienic conditions during hunting, transport, handling, and marketing may further increase the risk of contamination and transmission to consumers. In addition, information regarding the prevalence and molecular characteristics of NTS in bushmeat in Cameroon remains limited.

It is in this context that the assessment of the prevalence and molecular epidemiology of nontyphoidal serovars of *S. enterica*, particularly *Salmonella* ser. Enteritidis and *Salmonella* ser. Typhimurium, in bushmeat carcasses sold in markets in the Central Region of Cameroon, constitutes a major scientific and public health challenge.

## 2 Materials and Methods

### 2.1 Study Design and Collection Site

A cross‐sectional study was carried out in five localities of five divisions in the Central Region of Cameroon (Akonolinga, Nanga‐Eboko, Nkoabang, Yaoundé, and Mbalmayo) from March to October 2024 (Figure 1). A preliminary survey was first carried out to identify the various sites, markets, and points of sale for fresh bushmeat in the localities (Figure 1).

**Figure 1:**
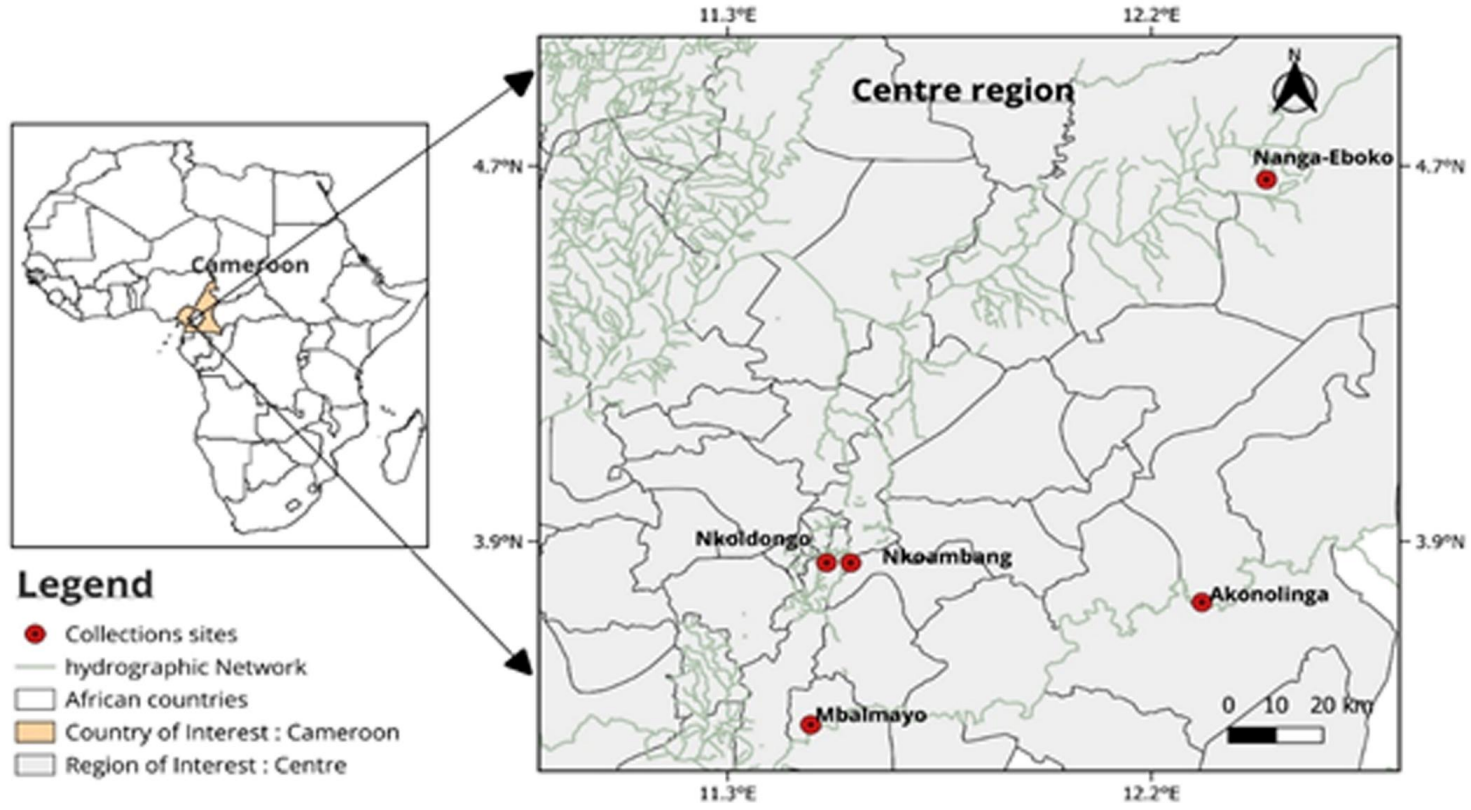
Map of the Central Region of Cameroon showing sites where bushmeat samples were collected.

### 2.2 Sample Collection

Meat carcasses were collected from species such as the porcupine (*Atherurus africanus*), the hedgehog (*Atelerix albiventris*), the pangolin (*Phataginus tricuspis*), the monitor lizard (*Varanus niloticus*), the hare (*Lepus victoriae*), the deer (*Cervus elaphus*), the civet (*Civettictis civetta*), the tiger cat (*Leopardus tigrinus*), and the wild boar (*Sus scrofa*). These species are widely consumed by local populations. Sites were selected based on their level of fresh bushmeat trade and accessibility.

The sample size was calculated using the Lorenz formula $n=\frac{z^{2}\cdot P\left(1-P\right)}{d^{2}}$ with the following parameters, *Z*(1 − *a*/2) as a standard normal variable (1.96) at 5%, assuming a *Salmonella* contamination prevalence of 10% reported by Kagambèga et al. (2013) in Burkina Faso on meat carcasses, with a type I error (*p* < 0.05) and a 95% confidence level. On the basis of the minimum sample size requirement, a total of 142 fresh bushmeat carcass samples (100–200 g each) were randomly collected from internal tissues of animals across five towns in five divisions of the Central Region of Cameroon. Samples were collected using individual sterile plastic bags and transported in a cooler to the Food and Drug Safety Laboratories Unit at the Biotechnology Center of the University of Yaoundé I. Isolation of bacteria was performed in accordance with the recommendations of International Organization for Standardization (ISO 2015).

### 2.3 Bacterial Isolation and Identification

Isolation of *Salmonella* spp. was performed as described by Bachand et al. (2012). Briefly, 25 g of pooled internal tissue from each carcass sample was added to 225 mL of buffered peptone water (Liofilchem S.r.l., Italy) and homogenized for 2 min using a Bag‐Mixer. After 24 h of incubation at 37°C, 1 mL of the pre‐enriched broth was transferred into 9 mL of Rappaport–Vassiliadis Soy broth (Liofilchem S.r.l., Italy) and incubated for an additional 24 h at 42°C. The inoculum was diluted serially, and 1 mL of each dilution was streaked onto a *Salmonella*‐Shigella agar plate (Liofilchem S.r.l., Italy) and incubated at 37°C for 24 h. Small gray colonies with black centers on *Salmonella*‐Shigella agar, presumptive for *Salmonella*, were subcultured onto nutrient agar plates for purification and incubated at 37°C for 24 h.

The following phenotypic tests: motility test, Gram stain, Catalase and oxidase tests (BD, Franklin Lakes, USA) were performed on the suspect isolates. Suspect isolates were grown on triple sugar iron (TSI) media (Liofilchem S.r.l., Italy) for sugar fermentation and urease testing. Isolates were then preserved in Brain Heart Infusion broth supplemented with 20% glycerol (BHI) (Liofilchem S.r.l., Italy), stored at −20°C for further analysis (Basuony et al. 2022).

### 2.4 Molecular Identification of *Salmonella* Isolates

#### 2.4.1 DNA Extraction

Bacterial DNA was extracted using the heat lysis method, following the protocol described by Ali et al. (2009) with minor modifications. Young bacterial colonies (18–24 h old) were grown on nutrient agar and incubated at 37°C for 24 h. After growth, three to four colonies were introduced into 1.5 mL Eppendorf tubes containing 500 μL of nuclease‐free water. The mixture was vortexed for 5 s, then heated at 100°C for 15 min and centrifuged at 12,000 rpm for 10 min. After extraction, DNA concentration and purity were assessed using a NanoDrop spectrophotometer. Finally, 200 µL of the supernatant was collected and stored at –20°C for subsequent analysis.

#### 2.4.2 Intra‐Serotyping Multiplex Polymerase Chain Reaction (m‐PCR) Characterization

m‐PCR was used for genus‐level detection of *Salmonella* spp. and for the specific identification of *Salmonella* ser. Enteritidis and *Salmonella* ser. Typhimurium serovars using InvA, IE‐1, and FliC‐C gene targets, respectively (Table 1). PCR reactions were performed in a total volume of 25 μL containing 12.5 μL of 2X One Taq Hot Start 2X Master Mix with standard buffer (New England Biolabs, MA, USA.), 2 μL of InvA primer, 2 μL of IE‐1 primer, 2 μL of Flic‐C primer, 5 μL of bacterial DNA, and 1.5 μL of nuclease‐free water. The m‐PCR protocol consisted of an initial denaturation step of 5 min at 94°C, followed by 35 cycles of 30 s at 94°C, 1 min at 58°C, and 1.5 min at 72°C, then a final extension step of 10 min at 72°C. PCR products were analyzed by 1.5% agarose gel electrophoresis, and the presence of amplicons at 796, 316, and 432 bp was indicative of the InvA, IE‐1, and Flic‐C genes, respectively (Basuony et al. 2022; Paião et al. 2013).

**Table 1:** Primers used to identify *Salmonella* spp. in the multiplex PCR and RAPD–PCR.

Test	Primers name	Target	Primer sequence	Product size (pb)	Reference
Multiplex PCR	InvA	*Salmonella* spp.	F: CGG TGG TTT TAA GCG TAC TCT T	796	Paião et al. (2013)
			R: CGA ATA TGC TCC ACA AGG TTA		
	IE‐1	*S*. Enteritidis	F: AGT GCC ATA CTT TTA ATG AC	316	
			R: ACT ATG TCG ATA CGG TGG G		
	Flic‐C	*S*. Typhimurium	F: CCCGCTTACAGGTGGACTAC	432	
			R: AGCGGGTTTTCGGTGGTTGT		
RAPD–PCR	P5		AACGCGCAAC		Hasan and Lafta (2021)

Abbreviations: PCR, polymerase chain reaction; RAPD, random amplification of polymorphic DNA.

### 2.5 Genetic Fingerprinting by Random Amplification of Polymorphic DNA (RAPD–PCR)

*Salmonella* ser. Enteritidis and *Salmonella* ser. Typhimurium strains previously confirmed by m‐PCR assay were subjected to RAPD–PCR as previously described by Hasan and Lafta (2021) with minor modifications. On the basis of the low difference in discriminatory efficacy they observed among primers P5, OPP‐16, and the five‐primer multiplex, we chose to work with primer P5. Briefly, the RAPD–PCR reaction was performed in a total volume of 25 µL containing 5 µL One Taq Hot Start 2X Master Mix with standard buffer (New England Biolabs, MA, USA.), 1 µL of the P5 oligonucleotide primer, 3 µL of bacterial DNA extract, and 16 µL of nuclease‐free water. The amplification conditions included an initial denaturation at 95°C for 5 min, followed by 35 cycles of denaturation at 94°C for 1 min, annealing at 40°C for 1 min, and extension at 72°C for 1 min. A final extension step was carried out at 72°C for 7 min, followed by a hold at 4°C. PCR products were separated by electrophoresis on a 1.5% agarose gel, visualized under UV light using a Biometra imaging system (Germany), and subsequently analyzed (Hasan and Lafta 2021).

### 2.6 Statistical Data Analysis

All data were compiled and analyzed using Microsoft Excel (Excel 2016, Los Angeles, CA, USA). Genetic fingerprints obtained from RAPD were first analyzed visually, and a positive response (a score of “1”) was defined as the presence of a visible band of a given size, while a negative response (a score of “0”) was defined as the absence of any band of the same size. These scores were then entered into a Microsoft Excel spreadsheet and into Phyltools software. The genotypic profiles generated by the RAPD–PCR methods were analyzed, and groupings were performed using the Unweighted Pair Group Method with Arithmetic Mean (UPGMA) and Jaccard's similarity coefficient. A dendrogram was constructed to assess the relatedness of *Salmonella* isolates using the genetic distance method in MEGA Version 12.0. Associations between the presence of *Salmonella* and categorical variables, notably the collection site and the species of bush meat, were assessed using the Chi‐square test. Where the expected frequencies were low, the test was applied. Statistical analyses were performed using R software version 4.4.1, with a significance level of *p* < 0.05.

## 3 Results

### 3.1 Phenotypic Characterization and *Salmonella* Prevalence

From the 142 bushmeat carcasses, 117 (82.39%) had colonies of presumptive *Salmonella* spp. These suspected colonies presented on *Salmonella*‐Shigella agar, with shiny, smooth surfaces that were opaque to slightly transparent. Most colonies appeared gray with a black center or were completely black, indicating they are hydrogen sulfide producers. Microscopic analysis disclosed Gram‐negative rod‐shaped bacteria. Biochemical identification via TSI agar revealed glucose fermentation. All the isolates were positive for catalase, negative for oxidase, and negative for urease. These phenotypic and biochemical traits aligned with the identification profile of presumptive *Salmonella* isolates.

### 3.2 Molecular Identification by m‐PCR

The 117 isolates phenotypically suspected as *Salmonella* spp. were subjected to m‐PCR. A sample of the Agarose gel electrophoresis indicating the presence ans absence of amplicons is shown in Figure 2. Among the 98 PCR‐confirmed *Salmonella* isolates, the InvA gene was detected in 48. Serotyping primers (Flic‐C and IE‐1) identified two zoonotic foodborne serotypes: *Salmonella* ser. Typhimurium (five isolates) and *Salmonella* ser. Enteritidis (93 isolates), corresponding to 3.52% and 65.49% of confirmed isolates, respectively (Table 2).

**Figure 2:**
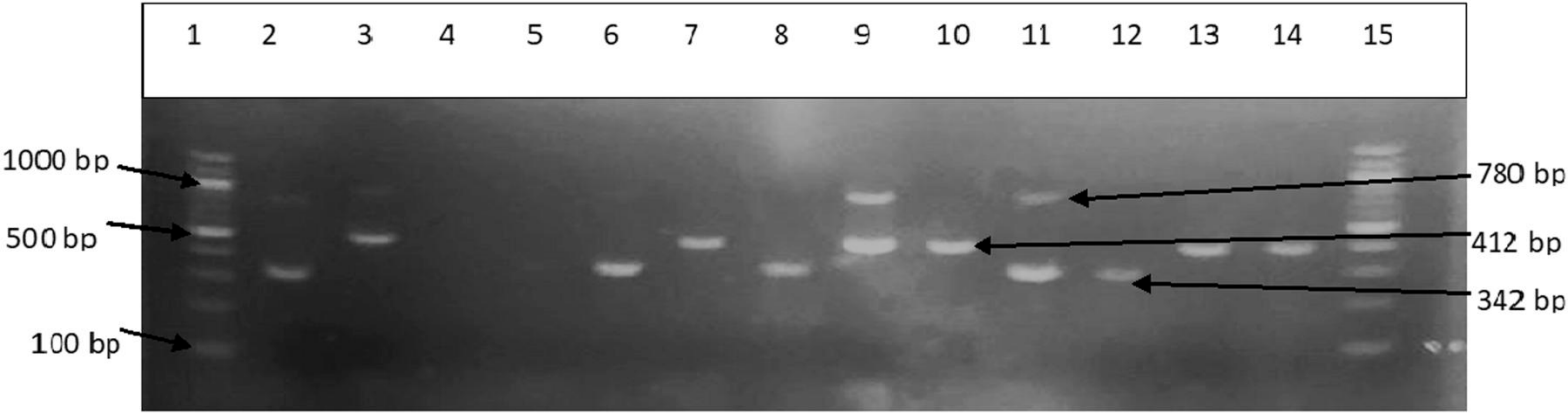
Agarose gel electrophoresis (1.5%) showing ~ 780 bp amplicons of the InvA gene characteristic of invasive *Salmonella enterica* subsp. isolates. Legend: Multiplex polymerase chain reaction amplification showing 412 bp fragments corresponding to the Flic‐C gene (*Salmonella* ser. Typhimurium) and 342 bp fragments corresponding to the IE‐1 gene (*S*. Enteritidis). Wells 1 and 15, molecular weight markers; well 2, *S*. Enteritidis positive control; well 3, *Salmonella* ser. Typhimurium positive control; well 4, negative control. Wells 6, 8, and 12: *Salmonella* ser. Enteritidis–positive samples lacking the invA gene. Well 11: *Salmonella* ser. Enteritidis–positive sample with the invA gene. Well 9: *Salmonella* ser. Typhimurium–positive sample with the InvA gene. Wells 10, 13, and 14: *Salmonella* ser. Typhimurium–positive samples lacking the InvA gene.

**Table 2:** Distribution of *Salmonella* serovars carrying the *invA* gene by bushmeat collection site.

Ville	Total sample collected	Presumptive *Salmonella* a	Total *Salmonella* spp.	*Salmonella* ser. Enteritidis^a^	*Salmonella* ser. Typhimurium^a^	InvA gene^a^
Akonolinga	31	24 (16.9%)	20	18 (58.06%)	2 (6.45%)	10 (32.26%)
Mbalmayo	32	33 (23.24%)	26	24 (64.86%)	2 (5.41%)	12 (32.43%)
Nanga‐Eboko	25	21 (14.79%)	21	21 (84.00%)	0 (0.00%)	7 (28.00%)
Nkoabang	24	20 (14.1%)	16	15 (62.50%)	1 (4.17%)	7 (29.17%)
Yaoundé	25	19 (13.39%)	15	15 (60.00%)	0 (0.00%)	9 (36.00%)
Total	142	117 (82.39%)	98	93 (65.49%)	5 (3.52%)	45 (31.69%)
Statistic analyze	*p* = 0.2892	*p* = 0.5537	*p* = 0.9776			

^a^ Percentage of positive samples within the collected animal species.

Of the 142 bushmeat carcasses collected from five sites in the Centre Region and categorized by animal species, 98 (69.01%) were found to be contaminated with NTS. The majority of isolates belonged to the serovar *S*. Enteritidis (65.49%), while *Salmonella* ser. Typhimurium accounted for 3.52% (Table 2). Prevalence of *Salmonella* ser. Enteritidis within the NTS varied among animal species, reaching 100% in hares, hinds, civets, and tiger cats, whereas no *Salmonella* strain was isolated from wild boar and tope carcasses (Table 3). The InvA virulence gene was identified in 45 isolates (45.91%), showing variable distribution across different host species and collection sites. However, the prevalence of InvA in isolated NTS was 4.08% in strains from Pangolin and Monitor lizards, and about 7.4% of strains from Hedgehogs, hares (3.06%), civet (6.12%), and 19.39% from Porcupines (Table 2).

**Table 3:** Frequency of bushmeat carcasses contaminated with *S*. Enteritidis and *S*. Typhimurium by animal species.

Species collected	Samples collected	Total *Salmonella* spp.^a^	*Salmonella* ser. Enteritidis^a^	*Salmonella* ser. Typhimurium^a^	Positive InvA gene
Hinds	3	3 (2.11%)	3 (100.00%)	0 (0.00%)	3 (100.00%)
Tiger cats	2	2 (1.4%)	1 (50.00%)	0 (0.00%)	0 (0.00%)
Civets	12	9 (6.34%)	9 (75.00%)	0 (0.00%)	6 (50.00%)
Hedgehogs	29	19 (13.38%)	18 (62.07%)	1 (3.45%)	7 (24.14%)
Hares	2	2 (1.4%)	2 (100.00%)	0 (0.00%)	2 (100.00%)
Pangolins	7	6 (4.22%)	5 (71.43%)	1 (14.29%)	4 (57.14%)
Porcupines	76	52 (36.7%)	49 (64.47%)	3 (3.95%)	19 (25.00%)
Wild boar	1	0 (00.00%)	0 (0.00%)	0 (0.00%)	0 (0.00%)
Tope	1	0 (00.00%)	0 (0.00%)	0 (0.00%)	0 (0.00%)
Monitor lizards	9	6 (4.22%)	6 (66.67%)	0 (0.00%)	4 (44.44%)
Total	142	98 (69.01%)	93 (65.49%)	5 (3.52%)	45
Statistic analyze			*p* = 0.5932	*p* = 0.9399	*p* = 0.0206

^a^ Percentage of positive samples within the collected animal species.

*Salmonella* ser. Enteritidis was isolated in all locations, with the highest frequency of 100% in samples from Nanga‐Eboko (21/21), 64.86% in Mbalmayo (24/37), 62.5% in Nkoabang (15/24), 60% in Yaoundé (15/25), and 58.06% in Akonolinga (18/31). In contrast, *Salmonella* ser. Typhimurium was detected at very low levels across all sites, with detection rates of 6.45% in samples from Akonolinga (2/31), 5.4% in samples from Mbalmayo (2/37), and 4.17% in samples from Nkoabang (1/24). However, no *Salmonella* ser. Typhimurium serovar was found in carcass samples from Yaoundé and Nanga‐Eboko (Table 3).

The Chi‐square test revealed no statistically significant correlation between animal species and the distribution of *Salmonella* ser. Enteritidis (*p* = 0.5932) and *Salmonella* ser. Typhimurium (*p* = 0.9399), as the *p* values were above the significance threshold of 0.05. Conversely, the data show a statistically significant association between the presence of the InvA gene and animal species (*p* = 0.0206), indicating that the distribution of this gene varies significantly across species. The statistical analysis did not reveal any significant association between the collection sites and the distribution of *Salmonella* ser. Enteritidis (*p* = 0.2892), *Salmonella* ser. Typhimurium (*p* = 0.5537) or the InvA gene (*p* = 0.9776). These results suggest a relatively homogeneous distribution of *Salmonella* serovars and the InvA gene across the different locations studied.

### 3.3 Molecular Typing Using the RAPD–PCR Method

DNA fingerprinting of *Salmonella* strains isolated from bushmeat carcasses by RAPD–PCR methods, using the P5 primer, revealed a diversity of distinct band profiles, with amplified fragments ranging in size from 100 to 1200 bp (Figure 3). This variation in profiles across strains confirms genetic differences among isolates, indicating a high degree of polymorphism among the *Salmonella* strains studied. Reproducibility of profiles and band resolution indicates efficient amplification with primer 5, enabling reliable genotype discrimination. The molecular weight marker well was used to estimate the relative sizes of the fragments. These results demonstrate the effectiveness of the RAPD‐P5 primer for assessing the genetic diversity of *Salmonella* strains of environmental origin.

**Figure 3:**
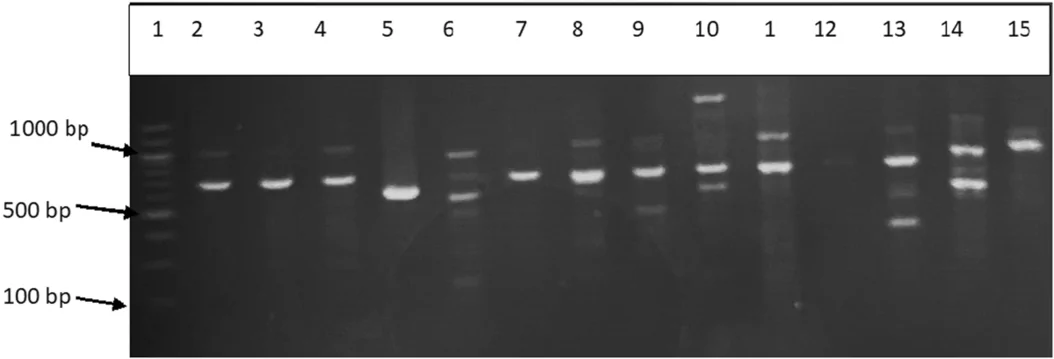
Representative RAPD agarose gel showing DNA fingerprints of NTS strains with the p5 primer on a 1.5% agarose gel. Legend: Lane 1, molecular weight marker; lanes 2–15, different bands of NTS strains isolated from bushmeat carcasses. NTS, nontyphoid *Salmonella*; RAPD, Random Amplification of Polymorphic DNA.

The dendrogram generated from *S*. Enteritidis serovars revealed genetic variation among the isolates, which were grouped into 06 distinct clusters based on an 80% similarity threshold (corresponding to a distance of 0.20) (Figure 4). This clustering pattern indicates moderate genetic diversity among the isolates. However, some exhibited a higher degree of similarity. Despite all isolates sharing at least 80% genetic similarity, the dendrogram suggests they may originate from a common source, a shared collection site, or share phenotypic characteristics.

**Figure 4:**
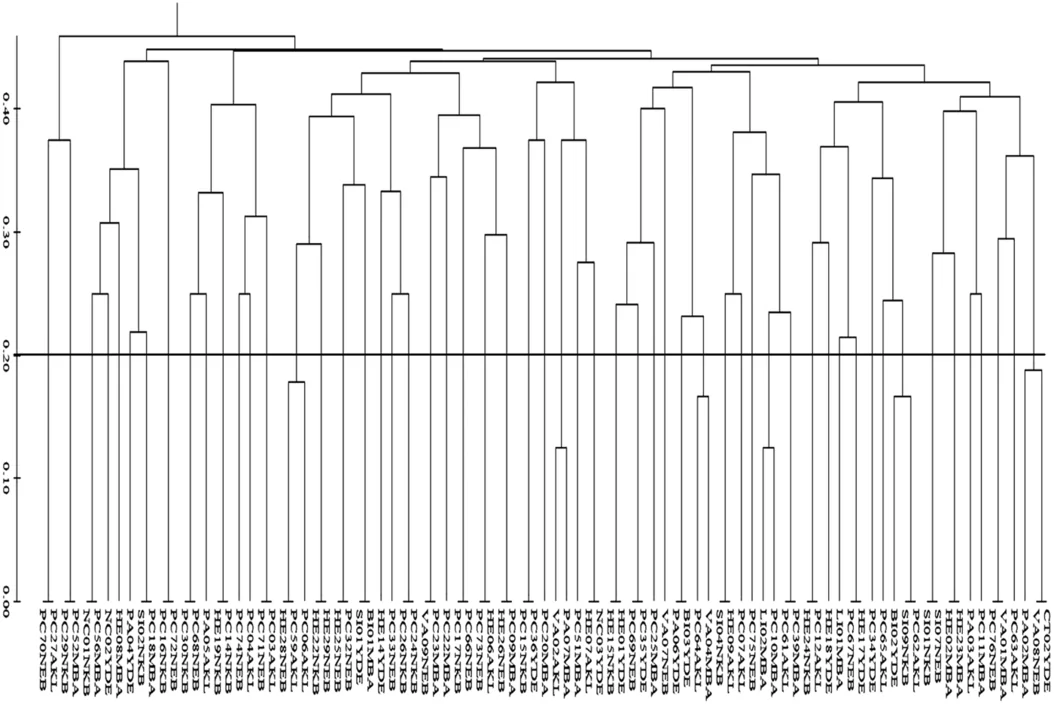
Dendrogram of the 93 *S*. Enteritidis strains, combined, constructed from pairwise comparisons of Jaccard similarity coefficients with grouping by UPGMA based on the presence or absence of amplified bands using MEGA X software. UPGMA, Unweighted Pair Group Algorithm.

The similarity dendrogram confirms this result. This shows that certain strains, although originating from different sites or carcasses, share very similar genetic profiles, likely linked to a common source of contamination or to clonal propagation of the same strain across multiple areas.

The dendrogram obtained from *S*. Typhimurium serovars (Figure 5) highlighted genetic differences among the strains, which were grouped into two related clusters at a 70% similarity level. By setting the similarity threshold to 80% (corresponding to a distance of 0.20), we obtain three distinct clusters at varying levels of similarity. Cluster 1 groups the isolates PC38MBA and PC28NKB; cluster 2 comprises the isolates HE06AKL and PA03AKL; and cluster 3 comprises the isolate PC01MBA only. This diversity may be associated with several factors, including the carcass collection site.

**Figure 5:**
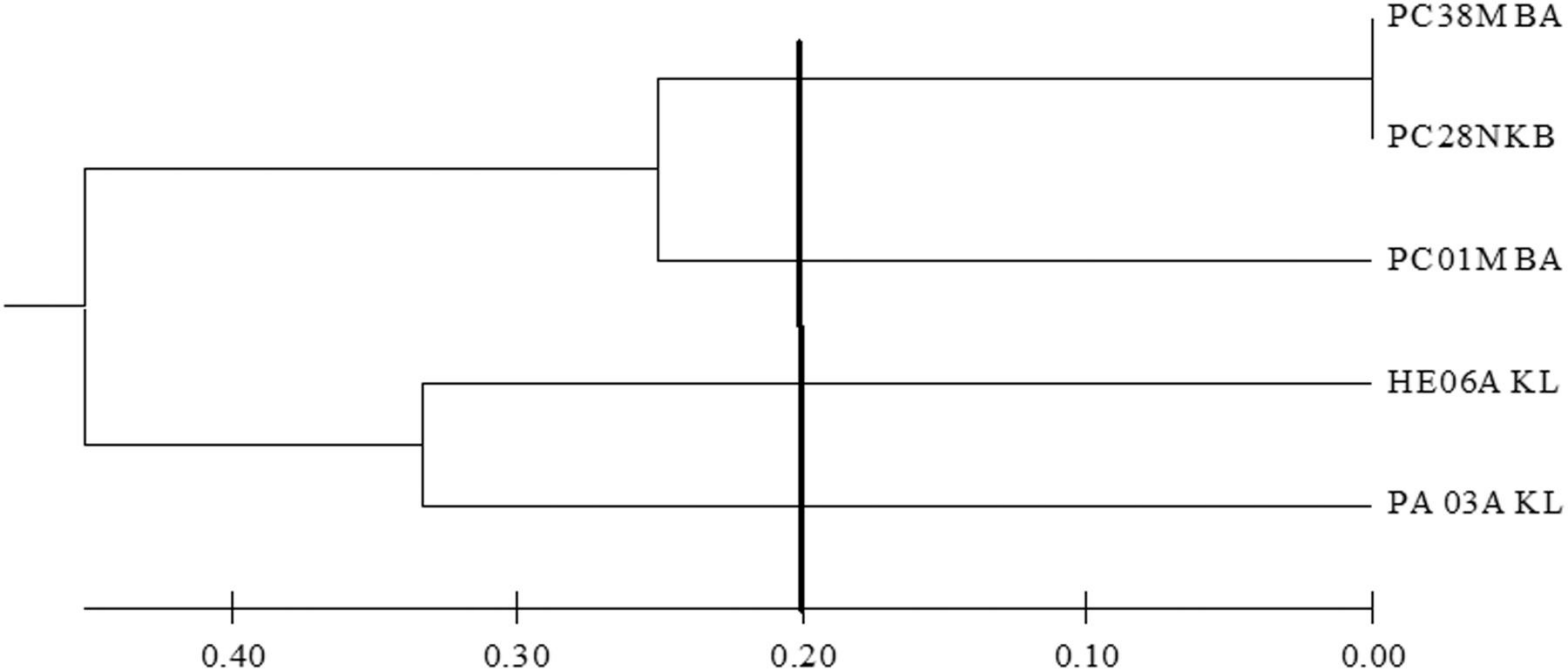
Dendrogram of the five combined *S*. Typhimurium isolates constructed from pairwise comparisons of Jaccard similarity coefficients with UPGMA clustering based on the presence or absence of amplified bands using MEGA X software. UPGMA, Unweighted Pair Group Algorithm.

## 4 Discussion

*Salmonella* is the leading cause of foodborne bacterial disease in humans worldwide. Infections linked to this pathogen rarely, if ever, lead to clinical disease in animals. However, the presence of the invasive gene in certain strains confers high virulence, enabling the pathogen to spread beyond the intestinal tract into the bloodstream. As a result, animal carcasses destined for human consumption are frequently contaminated during evisceration, and poor processing and preservation practices can lead to human transmission due to the high incidence of these pathogens in the animal intestinal tract. These factors are amplified when it comes to bushmeat, which is not subject to any official safety controls and is not sold under the protective laws that govern the hunting of these animals.

The results of this study indicate that *Salmonella* was isolated from 98 of 142 meat carcass samples, representing a prevalence of 69.01%. Although *Salmonella* is primarily a soil microorganism, its presence in bushmeat may result from environmental contamination or inadequate hygiene during processing and sale. Additionally, the studied wildlife often inhabits areas near human settlements and farms, increasing their exposure to potential sources of contamination. Environmental sources such as fecal matter from hunters, farmers, or domestic animals may further contribute to carcass contamination. Notably, the prevalence observed in this study is substantially higher than that reported by Bachand (2013) in Gabon, where only 0.8% of bushmeat tested positive, highlighting regional differences in sanitary practices. These findings underscore a significant public health concern, as consuming contaminated bushmeat may facilitate the transmission of *Salmonella* to humans, potentially leading to foodborne infections and associated health complications. The identification of *Salmonella* isolates at the genus level is generally done using the InvA gene, but a high specificity of IE‐1 and Flic‐C genes for the identification of *Salmonella* ser. Enteritidis and *Salmonella* ser. Typhimurium, respectively, was demonstrated by S. J. Wang and Yeh (2002). Khan et al. (2021) successfully used the Flic‐C gene alone to identify *Salmonella* ser. Typhimurium in poultry chicken, while Paião et al. (2013) combined InvA and IE‐1 or Flic‐C for the identification of these two serovars. In this regard, strains that lacked the InvA gene but demonstrated the presence of IE‐1 or Flic‐C genes were attributed to the genus since there is no proof that the two serovars are attributed to any other genus.

We found that *Salmonella* ser. Enteritidis in 65.49% (93/142) of samples and *Salmonella* ser. Typhimurium in 3.52% (05). *Salmonella* ser. Enteritidis and *Salmonella* ser. Typhimurium have different resistance capacities to environmental stress. In fact, H. Wang et al. (2020) demonstrated that *Salmonella* ser. Enteritidis showed the highest resistance to acidic, hyperosmotic, and oxidative stress compounds compared with *Salmonella* ser. Typhimurium and *Salmonella* ser. Infantidis. Moreover, Martin et al. (2026) also found enhanced environmental stress tolerance in *S. enterica* compared with other *S. enterica* serovars like *S*. Heidelberg, *S*. Newport, and *S*. Typhimurium. These findings can explain the high prevalence of *Salmonella* ser. Enteritidis or *Salmonella* ser. Typhimurium in our survey. This predominance is also reported by other authors. Indeed, our findings are consistent with previous systematic reviews conducted in 2014 and 2023 across Africa, which reported that *Salmonella* ser. Typhimurium and *Salmonella* ser. Enteritidis were the most prevalent serovars responsible for iNTS infections, accounting for over 90% of isolates (Hagedoorn et al. 2023; Uche et al. 2017). Additionally, Zou et al. (2012) reported that *Salmonella* ser. Enteritidis was the serovar most often implicated in human salmonellosis outbreaks. This level of prevalence differs from that reported by Shivaning Karabasanavar et al. (2020), who reported an overall *Salmonella* prevalence of 3.85% in poultry, of which 68.1% were *S*. Enteritidis and 31.8% were *S*. Typhimurium. Similarly, Thung et al. (2016) obtained prevalence of 20%, 6.7%, and 2.5%, respectively, for *Salmonella* spp., *Salmonella* ser. Enteritidis, and *Salmonella* ser. Typhimurium in samples of raw chicken meat sold in retail markets in Malaysia.

The InvA gene is a highly conserved virulence gene in the *Salmonella* genome that enables characterization (Buehler et al. 2019; Mthembu et al. 2019). It enables local invasion and is essential for bacterial entry into epithelial cells from the intestinal lumen (Bangera et al. 2018). Our study showed that out of 98 NTS, 45 (45.91%) isolates were positive for the InvA gene. The InvA gene is primarily transferred horizontally, and its high transfer frequency may be associated with its high abundance in the microorganism's growth environment. The InvA gene was highly prevalent in *Salmonella* ser. Enteritidis and *Salmonella* ser. Typhimurium NTS isolates, in 89.8% of reported clinical cases (Ohud et al. 2012), 99.5% of isolates from children, animals, and food (Dione et al. 2011), and 100% of poultry meat isolates (Nazari Moghadam et al. 2023), highlighting its role as a key virulence gene. Although many authors have described a high presence of InvA genes in *S. enterica* many others have observed that not all isolates identified express the InvA gene (Qadir et al. 2026; Abhadionmhen et al. 2023). The lower prevalence of the InvA gene in our *Salmonella* ser. Enteritidis and *Salmonella* ser. Typhimurium isolates may reflect limited exposure to *S. enterica* strains carrying this gene or loss due to environmental stress or mutations; in fact, Koochakzadeh et al. (2015) reported that all 10 *Salmonella* isolates from 103 wild captive herbivores in Iran harbored InvA. The presence of the InvA gene in these NTS strains should be considered as an alert from a public health perspective. Moreover, the absence of the InvA gene in some *Salmonella* isolates does not allow us to classify them as nonvirulent, as this is only one of the virulence factors observed in this genus. Nikiema et al. (2021) observed in human stool and sandwiches that some *Salmonella* isolates that did not express the InvA gene also expressed other virulent genes, like finA, stn, spvR, or spvC. In the absence of the InvA gene, the strain is limited only in its ability to invade. It is also important to consider that the absence of detection of a highly conserved gene can also be associated with very low expression, mutation, or even, in a few cases, protocol failure. In these regards, total genome sequencing is the best way to investigate these reasons.

Determining genetic variation between *Salmonella* spp. isolates is a crucial step for epidemiological surveillance, infection control, and outbreaks between circulating strains. The use of the RAPD fingerprinting technique is a promising, easy‐to‐use typing tool suitable for assessing genetic diversity in bacteria (Hasan and Lafta 2021; Jin et al. 2006). This method is based on the random amplification of genomic DNA fragments using arbitrarily designed short primer sequences of 9–10 nucleotides that anneal blindly to complementary sites throughout the complete genomic DNA of an organism (Shekhawat et al. 2019). Although we decided, for convenience, to use a single primer rather than a multiplex, our results confirm the widely observed diversity of *Salmonella* spp. Isolates in animal samples. In fact, these findings corroborate the extensive genetic diversity of *Salmonella* isolates reported in previous studies, including *S*. Typhi from Egypt (Rezk et al. 2012), *S. enterica* from food products across Egyptian regions (El‐Sharkawy et al. 2017), and poultry and beef isolates in India (Shekhawat et al. 2019), suggesting that such diversity may facilitate pathogen adaptation to different hosts and environments, thereby complicating surveillance and control efforts.

Our findings are also consistent with those of Hasan and Lafta (2021), who characterized *Salmonella* isolates from broilers and layers in Iraq using RAPD–PCR and reported considerable genetic diversity, with multiple clusters reflecting varied sources of introduction to farms. Similarly, Hasan and Lafta (2024) observed extensive diversity among *Salmonella* strains isolated from chicken, feed, and water, with similarity dendrograms revealing both inter‐ and intra‐matrix variations, indicative of multifactorial contamination. The similarity of outcomes between these studies and our results attests to the discriminatory power of RAPD–PCR in differentiating *Salmonella* strains across diverse food matrices. In our study, the observed clustering of isolates not only highlights multiple routes of contamination in bushmeat but also underscores critical implications for traceability, surveillance, and the implementation of targeted control measures to mitigate the spread of *Salmonella* within the population.

## 5 Conclusion

This study revealed that bushmeat carcasses sold in the Central Region of Cameroon were highly contaminated with *Salmonella* spp. Out of 142 samples, 98 (69.01%) tested positive. *S.* Enteritidis was the most common serotype among the isolates, accounting for 93/98 (94.90%). *Salmonella* ser. Typhimurium made up 5.10% (5/98) of the isolates. The InvA gene, which is associated with virulence, was detected in 45.92% (45/98) of nontyphoidal *Salmonella* isolates. This included 60% (3/5) of *Salmonella* ser. Typhimurium and 45.16% (42/93) of *S*. Enteritidis. This shows that the strains currently circulating could invade and cause disease. RAPD–PCR genetic characterization revealed substantial diversity within serotypes. Six genetic clusters were found among *Salmonella* ser. Enteritidis isolates and three among *Salmonella* ser. Typhimurium isolates. These results indicate the presence of genetically diverse *Salmonella* strains in the bushmeat distribution chain and validate the efficacy of RAPD–PCR as a rapid epidemiological typing method for strain differentiation. In general, the study shows that bushmeat could be a source and a route for pathogenic nontyphoidal *Salmonella* to reach people in Cameroon. The high level of contamination and the presence of virulent strains show that sanitary controls need to be stricter throughout the bushmeat supply chain, especially during transportation, handling, processing, and storage. These results will help advocate for the protection of wild animals and prevent hunting.

## Author Contributions

**Marius Dongmo:** conceptualization, investigation, writing – original draft, methodology, visualization, writing – review and editing, formal analysis, data curation. **Akindeh Mbuh Nji:** conceptualization, methodology, supervision, writing – review and editing, validation. **Rhoda Nsen Bughe:** investigation, writing – original draft, writing – review and editing, funding acquisition, visualization. **Calvino Fomboh Tah:** writing – review and editing, visualization, methodology, software, data curation, formal analysis. **Wilfred Fon Mbacham:** funding acquisition, writing – review and editing, validation, methodology, project administration, resources. **Sylvain Leroy Sado Kamdem:** conceptualization, funding acquisition, writing – original draft, validation, methodology, writing – review and editing, visualization, supervision, project administration, resources.

## Ethics Statement

This research was carried out as part of a PhD thesis in Food and Industrial Microbiology and was ethically approved by the Centre de Recherche et Formation Doctorale, Science de la Vie, Santé et Environnement (CRFD‐SVSE) of the University of Yaoundé I. The review of this research project and the sampling authorizations were approved by the Joint Institutional Review Board for Animal & Human Bioethics (JIRB) under approval reference number BTC‐JIRB2024‐114.

## Conflicts of Interest

The authors declare no conflicts of interest.

## Data Availability

This published article includes all the data generated or analyzed during this study.
